# ﻿*Hemipiliazhuxiensis* (Orchideae, Orchidaceae), a new species from Hubei Province, China

**DOI:** 10.3897/phytokeys.247.131618

**Published:** 2024-10-16

**Authors:** Cai-quan Shen, Gui-Hua Lu, Xi-Tang Chen, Li-Sha Yi, De-Qing Lan, Rui Qin, Hong Liu

**Affiliations:** 1 Hubei Provincial Key Laboratory for Protection and Application of Special Plant Germplasm in Wuling Area of China, Key Laboratory of State Ethnic Affairs Commission for Biological Technology, College of Life Sciences, South-Central Minzu University, Wuhan 430074, Hubei, China South-Central Minzu University Wuhan China; 2 Hubei Jiugongshan National Nature Reserve Administration, Xianning 437625, China Hubei Jiugongshan National Nature Reserve Administration Xianning China

**Keywords:** Morphology, phylogeny, subtribe Orchidinae, Zhuxi County

## Abstract

*Hemipiliazhuxiensis* (Orchidaceae), is a new species discovered in the Shibali Long Canyon National Nature Reserve, Zhuxi County, Hubei Province, China. It is morphologically similar to *Hemipiliahenryi* and *Hemipiliacrassicalcarata*, but differs in having an oblong, simple labellum with a slightly involute margin, an upcurved apex, and a spur shorter than the ovary. Molecular phylogenetic analyses, using nuclear (nrITS) and plastid (combined *matK*, *psaB*, *psbA-trnH*, *rbcL* and *trnL-F*) DNA sequences, confirm that *H.zhuxiensis* is closely related to *Hemipiliahenryi* and *Hemipiliacrassicalcarata*, supporting its recognition as a new species in the H.sectionHemipilia as defined by Tang et al. (2015).

## ﻿Introduction

The genus *Hemipilia* Lindley *sensu stricto* (subtribe Orchidinae, Orchidaceae) comprises c. 13 species (Chase et al. 2015). *Hemipilias.s.* is characterized by distinct morphological features, such as protruding and tongue-like rostellum, separate stigma and rostellum, basal and single leaves (Luo and Chen 2000; Chen et al. 2009). Nevertheless, the phylogenetic relationships between *Hemipilia* and other genera within the subtribe Orchidinae remain controversial (Jin et al. 2014, 2017; Tang et al. 2015). The monophyletic *Hemipilias.s.* is clustered with *Ponerorchisbrevicalcarata* (Finet 1901: 420) von Soó (1966: 353) and *Hemipiliopsispurpureopunctata* (Lang in Lang and Ji 1978: 127) Luo and Chen (2003: 450) in a strongly supported clade (Luo 1999; Bateman et al. 2003; Jin et al. 2014).Tang et al. (2015) further proposed a broad circumscription of *Hemipiliasensu latissimo*, lumping *Neottianthe* Schltr., *Ponerorchis*, *Tsaiorchis* Tang & Wang and *Hemipiliopsis* into a single monophyletic genus. Most recently, Jin et al. (2017) updated the phylogeny with samples from the subtribe Orchidinae and recognized *Hemipilia*, *Ponerorchis*, and *Tsaiorchis* as distinct genera. Therefore, the circumscription of *Hemipilia* still requires refinement with additional data.

In recent years, many new species of *Hemipilia* have been discovered (Tang et al. 2016; Wang et al. 2022; Yang et al. 2022). During our 2020 field investigation in the Wuling Mountains of Hubei Province, we found numerous small, purple-flowered *Hemipilia* species. However, the literature shows no such characteristics in any of *Hemipilia* species studied before. The oblong and simple labellum with a slightly involute margin and a shorter spur distinguishes it from all the known species of *Hemipilia*. Morphological and phylogenetic analyses suggest that *H.zhuxiensis* is closely related to *H.henryi* and *H.crassicalcarata*. Consequently, we describe it as a new species in H.sect.Hemipilia as defined by Tang et al. (2015).

## ﻿Materials and methods

### ﻿Morphological observations

The morphological characterization and description of the new species are based on the comprehensive examination of both living plants and the herbarium specimens. The voucher specimens of *Hemipiliazhuxiensis* and *Hemipiliahenryi*, collected from Zhuxi County in Hubei Province, have been deposited in the Wuhan Botanical Garden, CAS (HIB). The list of all herbarium specimens examined for this study is provided in Appendix 1.

### ﻿Phylogenetic reconstruction

Molecular analysis was performed using 89 samples (including 2 newly sequenced) and 6 DNA sequence makers (nrITS, *matK*, *psaB*, *psbA-trnH*, *rbcL*, *trnL-F*) to explore the phylogenetic placement of the new species within Orchidinae following the phylogenetic study of Jin et al. (2017) and Yang et al. (2022). Three species (*Coryciumingeanum*, *Coryciumnigrescens*, and *Ceratandragrandiflora*) were considered as outgroups following Yang et al. (2022).

DNA of *Hemipiliazhuxiensis* and *Hemipiliahenryi* was extracted from silica-dried leaf fragments using the modified 2×CTAB procedure of Doyle and Doyle (1987). All sequences were obtained from the genome skimming data. DNA extraction, library preparation, and sequencing were performed at MajorBio Company (Shanghai, China). The nrDNA regions (18S-ITS1-5.8S-ITS2-26S) and complete chloroplast genome were assembled using GetOrganelle v1.7.4 with default parameters (Jin et al. 2020). Chloroplast genome annotation was performed with Geseq (Tillich et al. 2017). Annotation results were checked, adjusted and used to extract DNA sequence makers accordingly (nrITS, *matK*, *psaB*, *psbA-trnH*, *rbcL*, *trnL-F*) in Geneious 11.0.4 (Kearse et al. 2012). The final nrITS and plastome sequences of *Hemipiliazhuxiensis* and *Hemipiliahenryi* have been submitted to GenBank, and their accession numbers are provided in Table 1.

**Table 1.** GenBank accession numbers of taxa included in phylogenetic reconstruction. Sequences generated in this study are marked with asterisks (*). Missing data are indicated with “–”.

Species	ITS	*matK*	*psaB*	*psbA_trnH*	*rbcL*	*trnL_F*
*Hemipiliazhuxiensis*	PP988699	PP999314	PP999314	PP999314	PP999314	PP999314
*Hemipiliahenryi*	PP988698	PP999315	PP999315	PP999315	PP999315	PP999315
*Hemipiliaalpestris*	KJ460093	KJ452849	MF944593	MF944800	KJ451547	MF945360
*Hemipiliaamplexifolia*	KM651222	KM651386	–	KM651467	–	KM651546
*Hemipiliabasifoliata*	KM651223	KM651387	–	KM651468	–	KM651547
*Hemipiliacapitata*	KM651224	KM651388	–	KM651469	–	KM651548
Hemipiliacf.faberi	KM651226	KM651395	–	KM651471	–	KM651550
*Hemipiliafaberi*	KM651230	KM651389	–	KM651475	–	KM651554
*Hemipiliafarreri*	KJ460047	KJ452803	MF944558	MF944765	KJ451501	MF945325
*Hemipiliagonggashanica*	KM651233	KM651394	–	KM651478	–	KM651557
*Hemipiliagracilis*	KJ460036	JN696435	MF944434	MF944644	JN696420	MF945203
*Hemipiliahemipilioides*	KM651238	KM651400	–	KM651483	–	KM651562
*Hemipiliakeiskei*	KM651239	KM651401	–	–	–	KM651563
*Hemipiliakeiskeoides*	KM651240	KM651402	MF944552	KM651484	–	KM651564
*Hemipiliakinoshitai*	KM651241	KM651403	–	KM651485	–	KM651565
*Hemipilialepida*	KM651242	KM651404	–	KM651486	–	KM651566
*Hemipiliamonantha*	KJ460037	JN696436	MF944443	MF944653	JN696421	MF945212
*Hemipiliaphysoceras*	KM651246	KM651408	–	KM651490	–	KM651570
*Hemipiliaparceflora*	KJ460052	KJ452808	MF944562	MF944769	KJ451506	MF945329
*Hemipiliaphysoceras*	KM651247	KM651409	–	KM651492	–	KM651572
*Hemipiliathailandica*	KM651256	KM651419	–	KM651501	–	KM651581
*Hemipiliatrifurcata*	KJ460055	KJ452811	MF944565	MF944772	KJ451509	MF945332
*Hemipiliawenshanensis*	KM651258	KM651422	–	KM651504	–	KM651584
*Anacamptislaxiflora*	AM711747	KF997312	–	AM711707	KF997401	–
*Anacamptispyramidalis*	AY364870	JN894348	–	–	JN891189	KU931755
*Benthamiaperularioides*	MT500652	MT533554	–	–	MT506429	MT507741
*Brachycorythishenryi*	MF944262	MF945438	MF944465	MF944675	MF944873	MF945234
*Brachycorythisobcordata*	MF944263	MF945500	MF944533	MF944742	MF944936	MF945301
*Ceratandragrandiflora*	EU687530	EU687535	–	–	–	EU687540
*Chamorchisalpina*	–	FR832740	–	–	FN870786	–
*Coryciumingeanum*	EU301446	EU301499	–	–	–	EU301552
*Coryciumnigrescens*	EU301461	EU301514	–	–	–	EU301567
*Dactylorhizafuchsii*	MF944265	MF945400	MF944423	MF944633	MF944836	MF945192
*Dactylorhizaviridis*	JN696446	KJ452797	MF944555	MF944762	KJ451495	MF945322
*Galearisroborowskyi*	KM651265	KM651429	–	KM651511	–	KM651591
*Galearisspathulata*	KJ460094	KJ452850	MF944594	MF944801	KJ451548	MF945361
*Galearistschiliensis*	KJ460057	KJ452813	MF944566	MF944773	KJ451511	MF945333
*Galeariswardii*	MF944274	MF945417	MF944442	MF944652	MF944853	MF945211
*Hemipiliacalophylla*	KJ460095	KJ452852	MF944596	MF944803	KJ451550	MF945363
*Hemipiliacordifolia*	MF944329	MF945454	MF944481	MF944691	MF944888	MF945250
*Hemipiliacrassicalcarata*	KM651270	KM651434	–	–	–	KM651596
*Hemipiliacruciata*	MF944330	MF945462	MF944490	MF944700	MF944896	MF945259
*Hemipiliaflabellata*	KM651271	KJ452806	–	–	KJ451504	KM651597
*Hemipiliaforrestii*	KJ460049	KJ452805	MF944559	MF944766	KJ451503	MF945326
*Hemipiliagaleata*	KT183499	KT183498	–	–	–	KT183500
*Hemipiliakwangsiensis*	KM651272	KJ452851	MF944595	MF944802	KJ451549	MF945362
*Hemipiliayajiangensis*	OM009240	OM009241	OM009241	OM009241	OM009241	OM009241
*Hemipiliaavisoides*	OP597820	OP595696				OP595697
*Hemipiliapurpureopunctata*	KJ460051	KJ452807	MF944561	MF944768	KJ451505	MF945328
*Herminiumesquirolii*	KR350147	KR350183	KR350222	KR350277	KR350328	KR350367
*Himantoglossumhircinum*	AY351385	KF997261	–	–	KF997440	–
*Neolindleyacamtschatica*	KT338754	KF262003	–	KF262121	KF296612	–
*Neotineamaculata*	AM711744	–	–	AM711706	FN870882	KU931823
*Hemipiliacompacta*	JN696455	KJ452796	MF944554	MF944761	KJ451494	MF945321
*Hemipiliacucullata*	JN696456	KJ452792	MF944550	KM651522	KJ451490	MF945317
*Hemipiliafujisanensis*	KM651280	KM651444	–	KM651524	–	KM651606
*Hemipiliacucullata*	JN696454	KJ452791	MF944549	MF944756	KJ451489	MF945316
*Ophrysapifera*	AJ539529	AJ543953	AY381047	AM711642	AF074202	AJ409432
*Ophrysinsectifera*	MF944348	MF945396	MF944525	MF944734	MF944928	MF945293
*Orchisanthropophora*	AY364869	–	–	–	KF997307	EU294186
*Orchismascula*	AY351379	JN895683	–	HG800547	MK925129	KU931823
*Orchismilitaris*	AY014548	KF997352	–	–	KF997273	AY014586
*Orchispurpurea*	AY364882	–	–	–	KF997502	–
*Platantherabakeriana*	KJ460061	KJ452817	MF944569	MF944776	KJ451515	MF945336
*Hemipiliabasifoliata*	MF944399	MF945455	MF944482	MF944692	MF944889	MF945251
*Hemipiliabrevicalcarata*	KJ460041	KJ452793	MF944551	MF944758	KJ451491	MF945318
*Hemipiliacamptoceras*	MF944400	MF945409	MF944433	MF944643	MF944845	MF945202
Hemipiliacf.hui	KM651296	KM651462	–	KM651539	–	KM651621
*Hemipiliachidori*	KM651286	KM651450	–	KM651531	–	KM651613
*Hemipiliachusua*	MF944401	MF945460	MF944488	MF944698	MF944894	MF945257
*Hemipiliacucullata*	MF944402	MF945451	MF944477	MF944687	MF944885	MF945246
*Hemipiliagraminifolia*	KM651292	KM651456	–	KM651538	–	KM651620
*Hemipiliakiraishiensis*	MF944403	MF945445	MF944472	–	MF944879	MF945241
*Hemipiliahui*	MF944398	MF945425	MF944451	MF944661	MF944861	MF945220
*Hemipiliachusua*	MF944404	MF945475	MF944504	MF944713	MF944908	MF945273
*Hemipiliaoblonga*	MF944405	MF945472	MF944501	MF944710	MF944906	MF945270
*Hemipiliaomeishanica*	KM651299	KM651464	–	KM651542	–	KM651624
*Hemipiliacompacta*	MF944406	MF945458	MF944485	MF944695	MF944892	MF945254
*Hemipiliasichuanica*	KJ460059	KJ452815	MF944567	MF944774	KJ451513	MF945334
*Hemipiliasimplex*	MF944407	MF945427	MF944453	MF944663	MF944863	MF945222
*Hemipiliagraminifolia var. suzukiana*	KM651300	KM651459	–	KM651543	–	KM651625
*Hemipiliatetraloba*	MF944411	MF945440	MF944467	MF944677	MF944875	MF945236
*Hemipiliatibetica*	MF944412	MF945449	MF944476	MF944685	MF944883	MF945245
*Pseudorchisalbida*	KU974068	–	–	–	KF997412	GQ245349
*Pseudorchisstraminea*	DQ022894	–	–	–	FN870908	–
*Schizochilusflexuosus*	MT500598	FR832831	–	–	FN870929	MT507689
*Hemipiliapinguicula*	MF944417	MF945495	MF944528	MF944737	MF944931	MF945296
*Sirindhorniapulchella*	KJ460045	KJ452801	MF944557	MF944764	KJ451499	MF945324
*Steveniellasatyrioides*	AM711746	FR832840	–	–	–	KU931833
*Traunsteineraglobosa*	KT318279	–	–	HG800585	HG417055	–

“–” indicates lacking data.

Phylogenetic analyses were conducted using Maximum likelihood (ML) and Bayesian Inference (BI). All DNA sequence markers were aligned individually using MAFFT (Katoh and Standley 2013). Subsequently, the aligned sequences were manually adjusted and modified using trimAI (Capella-Gutiérrez et al. 2009). The concatenation of five plastid DNA sequences and the construction of a phylogenetic tree were eventually completed using PhyloSuite (Zhang et al. 2020). Nuclear and plastid data were analyzed separately following Tang et al. (2015). The best-fit DNA substitution model was estimated for nrITS using ModelFinder (Kalyaanamoorthy et al. 2017) and for the concatenated 5 plastid DNA sequences using PartitionFinder2 (Lanfear et al. 2016). The ML phylogenetic tree was obtained using IQ-TREE with ultrafast bootstrap support of 1000 replicates (Nguyen et al. 2015). The BI tree was constructed using MrBayes version 3.2.6 (Ronquist et al. 2012) with the Markov Chain Monte Carlo (MCMC) method and sampled every 1000 generations of a total of 2 million. Once the average standard deviation of split frequencies fell below 0.01, the first 25% of generated trees were discarded as a burn-in process, and the runs were considered to have reached a stable state. The phylogenetic trees were edited and visually optimized using TreeGraph2 (Stover and Muller 2010).

## ﻿Results

### ﻿Morphological comparison

In Hemipiliasect.Hemipilia, many species exhibit morphological similarity, characterized by relatively small purplish-red flowers, tongue-like rostellum, and ovate leaves with purple spots. We have selected *H.henryi and H.crassicalcarata* for morphological comparison with *H.zhuxiensis*, as they share general attributes, and also have the closest phylogenetic relationship. Morphological comparisons of *H.zhuxiensis*, such as leaf and flower characteristics, with the similar taxa *H.henryi and H.crassicalcarata*, are provided in Table 2. Morphological data are summarized from the literature (Chen et al. 2009) and recent observations of herbarium specimens (see Appendix 1).

**Table 2.** Morphological comparison of *H.zhuxiensis*, *H.henryi*, *H.crassicalcarata*.

Characters	*H.zhuxiensis*	*H.henryi*	*H.crassicalcarata*
**Numbers of leaves**	1	1	1
**Leaf shape**	Ovate	ovate	ovate to ovate-cordate
**Leaf color (adaxial)**	green with purple spots	green with purple spots	uniformly green
**Inflorescence length**	14–23 cm	17–30 cm	13–30 cm
**pedicel plus ovary long**	13–21 mm	16–24 mm	12–18 mm
**Petal shape**	obliquely ovate	obliquely rhombic-ovate	oblong-ovate, oblique
**Labellum shape**	oblong, margin slightly involute, apex upcurved	broadly obovate-cuneate	suboblong, margin irregularly crenate, apex truncate
**Labellum size**	10 × 3–5 mm	12 × 10 mm	13 × 9–10 mm
**Labellum margin**	Simple	3-lobed	simple
**Spur shape**	short and infundibuliform, apex hooked	straight and horizontal or slightly curved downward	straight and horizontal or sometimes slightly curved downward
**Spur shape**	narrowly conic	narrowly conic, gradually attenuate	cylindric, uniformly thick (not attenuate)
**Spur length**	4–6 mm, significantly shorter than ovary	14–18 mm, slightly shorter than ovary	10–12 mm, slightly shorter than ovary

### ﻿Phylogenetic reconstruction

**Figure 1.**
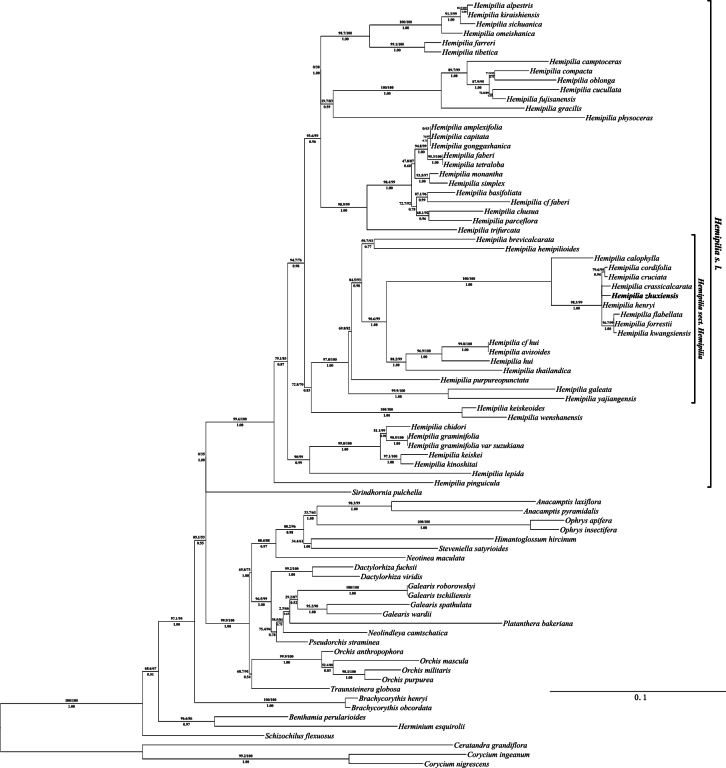
Phylogenetic placement of *H.zhuxiensis* (bold representation) using the maximum likelihood (ML) method based on nrITS. The maximum likelihood SH-aLRT supports and UFBoot supports (SH-aLRT_ML_ /UFboot_ML_) are displayed above the branches, and Bayesian posterior probabilities (PP_BI_) are displayed below the branches. Only SH-aLRT >= 80% and UFboot >= 95%, PP ≥ 0.95 are considered as strong supports.

**Figure 2.**
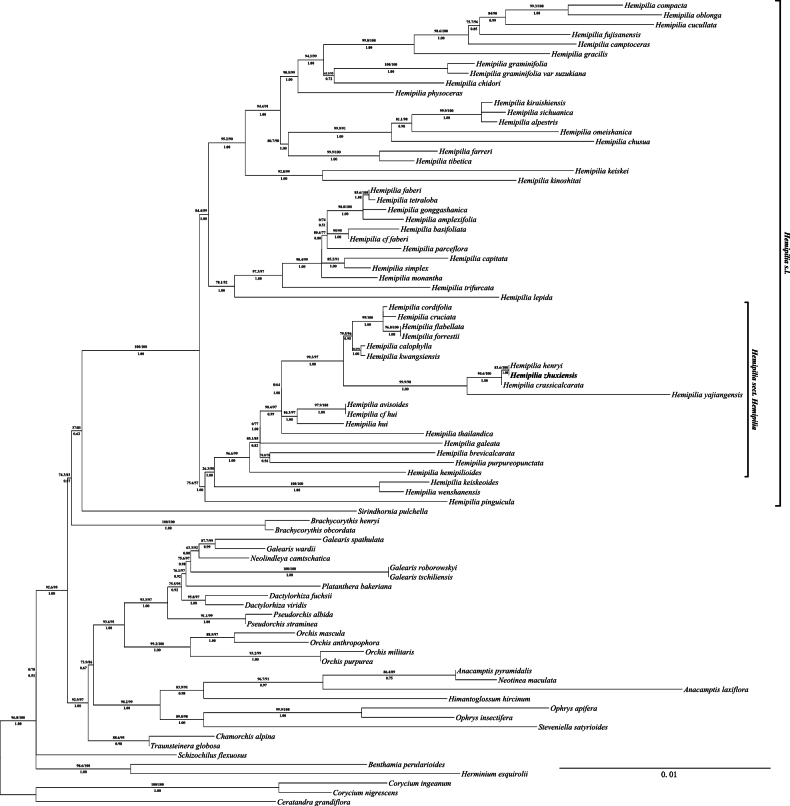
Phylogenetic placement of *H.zhuxiensis* (bold representation) using the maximum likelihood (ML) method based on the combined plastid DNA (*matK*, *psaB*, *psbA-trnH*, *rbcL*, *trnL-F*). The maximum likelihood SH-aLRT supports and UFBoot supports (SH-aLRT_ML_ /UFboot_ML_) are displayed above the branches, and Bayesian posterior probabilities (PP_BI_) are displayed below the branches. Only SH-aLRT >= 80% and UFboot >= 95%, PP ≥ 0.95 are considered as strong supports.

The phylogenetic relationship reconstructed based on nrITS and combined plastid datasets in this study, show minor differences (Figs 1, 2). Phylogenetic analyses based on nrITS data demonstrated that *H.zhuxiensis* does not cluster well with *H.crassicalcarata* and *H.henryi* into a clade. However, the plastid tree demonstrated that *H.zhuxiensis* is clustered with *H.crassicalcarata* and *H.henryi* into a clade with strong support (94.6/100/1, 83.6/100/1). These results indicate that *H.zhuxiensis* possibly has a close phylogenetic relationship with *H.crassicalcarata* and *H.henryi*, and analysis of data from nrITS and plastid trees consistently support *H.zhuxiensis* as a member of H.sect.Hemipilia.

### ﻿Taxonomic treatment

#### ﻿ Hemipilia zhuxiensis

Taxon classificationPlantaeAsparagalesOrchidaceae

Hong Liu sp. nov.

B850788F-2DC9-55F8-96BE-AC61B8ED3D5D

urn:lsid:ipni.org:names:77350375-1

Figs 3 , 4 , 5

##### Type.

China. • Hubei: Zhuxi County, Shibali Long Canyon National Nature Reserve; 733 m; 18 June 2020; *HSN13099* (holotype: HSN). To protect this species, the exact latitude and longitude are not published.

##### Diagnosis.

Though apparently similar to *H.henryi* Rolfe and *H.crassicalcarata* S.S.Chien, *H.zhuxiensis* shows certain differences in having ovate, purple-spotted leaf; 10 × 3–5 mm, oblong, simple labellum; slightly involute labellum margin; upcurved labellum apex; and a significantly shorter spur compared with the ovary (Table 2).

##### Description.

Terrestrial herbs, 17–25 cm tall. Tubers ellipsoid, 4–11 × 3–5 mm. Stem slender with 1 tubular cataphyll at the base, 1- or rarely 2-leaved. Leaf solitary, ovate, 6–12 × 5–8 cm, apex subacute, base cordate or contracted into amplexicaul sheath, adaxially green with purple spots, rarely uniformly green, abaxially pale green. Inflorescence terminal, 14–23 cm long with 1–2 sterile bracts; laxly 4–9-flowered; floral bracts lanceolate, to ca. 11 mm, apex acuminate or long acuminate. Flowers purplish red; pedicel and ovary straight or slightly arcuate, 13–21 mm long. Dorsal sepal ovate-elliptic, 6–9 × 3–7 mm, apex obtuse, 1-veined; lateral sepals broadly ovate, oblique, spreading, 7–10 × 5–8 mm, 1-veined, apex obtuse. Petals obliquely ovate, 6–7 × 4–5 mm, 1-veined, apex obtuse, purplish red. Labellum oblong, 10 × 3–5 mm, purplish red, adaxially finely papillate, simple; margin slightly involute, irregularly crenate; apex upcurved, obtuse to emarginate; spur short and infundibuliform, slightly curved downwards, narrowly conic, 4–6 mm long, entrance 2–2.5 mm wide. Column ca. 3 mm long; rostellum tongue-like, purple, ca. 2 mm, apex rounded.

**Figure 3.**
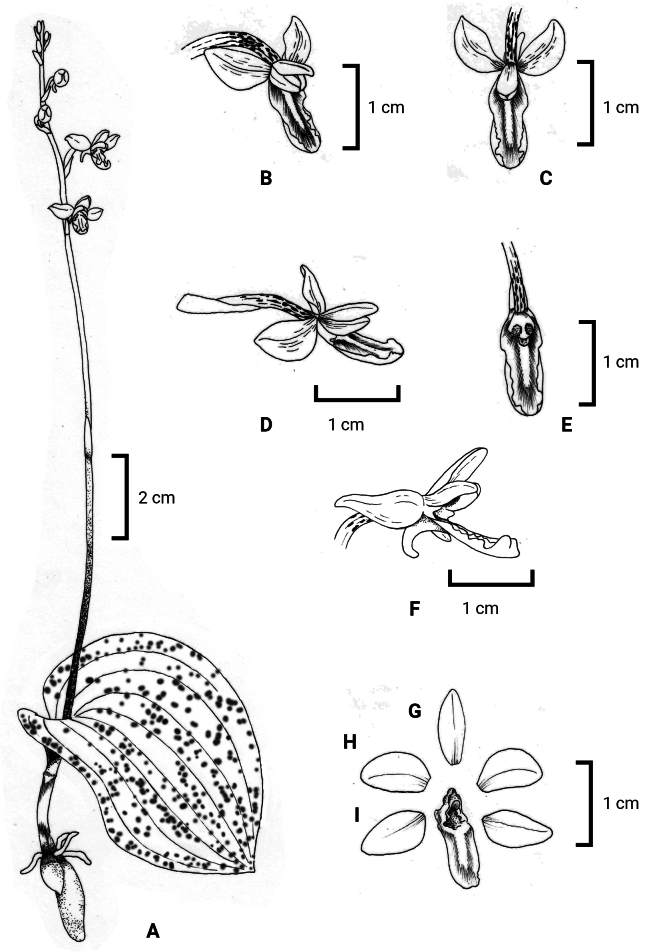
*Hemipiliazhuxiensis***A** habit **B** flower (front view) **C** flower (top view) **D** flower (side view) **E** labellum and column **F** spur **G** dorsal sepal **H** lateral sepals **I** petals. Drawn by Ta-Li Cai.

##### Distribution and habitat.

*H.zhuxiensis* is currently known to have two populations in Shibali Long Canyon National Nature Reserve, Zhuxi county, Hubei Province, China. The two populations are about 500 meters apart along the rock wall of the canyon. The new species grows on the rock wall together with *H.henryi*. The canyon is an arid valley, and many shrubs and mosses grow on the rock walls on both sides.

**Figure 4.**
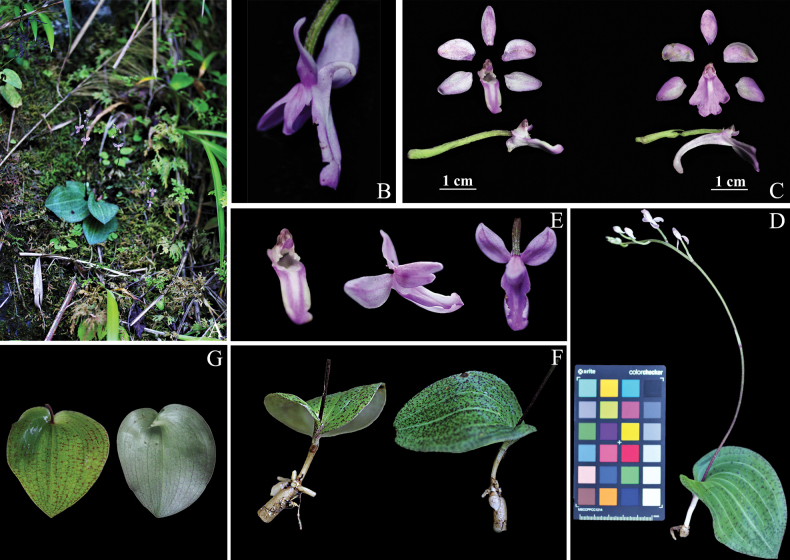
*Hemipiliazhuxiensis***A** habit **B** flower and spur (side view) **C** morphological contrast of *H.zhuxiensis* (left) and *H.henryi* (right) **D** flowering whole plant **E** column and labellum **F** tubers and roots **G** leaves (adaxial and abaxial view).

##### Preliminary conservation assessment.

Only two populations comprising approximately 10 mature individuals were found in Shibali Long Canyon National Nature Reserve, Zhuxi County, Hubei Province, China. The two populations are about 500 meters apart and growing on the rock wall alongside *H.henryi*. The habitat of *H.zhuxiensis* could be easily disturbed by development as it is close to roads and villages. Due to the limited population size and restricted distribution of *Hemipiliazhuxiensis*, the new species should be preliminarily classiﬁed as Critically Endangered (CR B2ab;C2a(i);D) according to the guidelines of the International Union for Conservation of Nature (IUCN) Red List Categories and Criteria (IUCN Standards and Petitions Committee 2022).

**Figure 5.**
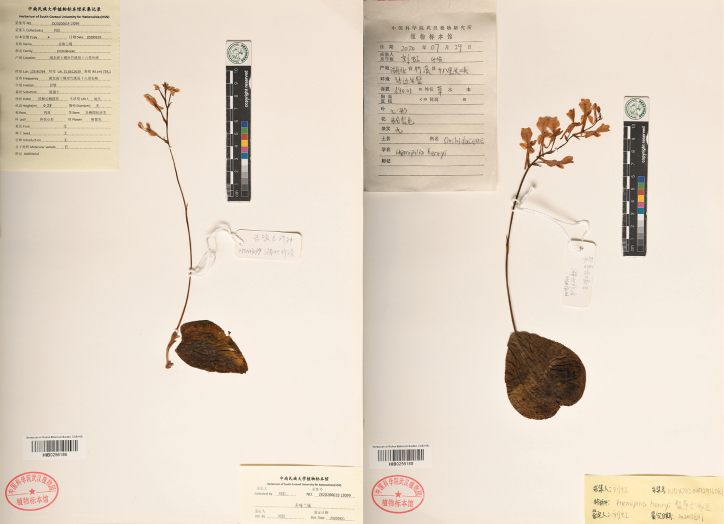
Photograph of the herbarium specimens of *H.zhuxiensis* Hong Liu (left) and *H.henryi* Rolfe (right).

##### Etymology.

The specific epithet refers to the name of the type locality in Zhuxi County.

##### Vernacular name.

The Chinese name is “竹溪舌喙兰”.

##### Phenology.

Flowering in June.

## ﻿Discussion

Molecular and morphological evidence demonstrates that *Hemipiliazhuxiensis* is a member of Hemipiliasect.Hemipilia (Tang et al. 2015). Morphologically, *H.zhuxiensis* is very similar to *H.henryi* and *H.crassicalcarata*, and it is sympatric with *H.henryi.* Therefore, we cannot exclude the possibility that *H.zhuxiensis* is a teratological form of *H.henryi.* However, it is clearly distinguishable by its distinctive labellum and spur. In addition, *H.zhuxiensis* has the earliest anthesis (June) compared to *H.henryi* (August) and *H.crassicalcarata* (July).

The phylogenetic trees based on nuclear and plastid DNA sequences show slight differences, but both datasets undoubtedly place the new species within the sect. Hemipilia according to Tang et al. (2015). The ITS-based phylogenetic tree shows that *H.zhuxiensis* cannot cluster well with *H.crassicalcarata* and *H.henryi* into a clade, likely due to the short ITS sequence and limited informative sites. In contrast, the plastid-based phylogenetic tree strongly supports the clustering of *H.zhuxiensis* with *H.henryi*, and further reveals a well-supported clade comprising these two species and *H.crassicalcarata*. These results indicate that *H.zhuxiensis* is closely related to *H.henryi* and *H.crassicalcarata.* In contrast with previous studies, while our study expands and reconstructs the phylogenetic tree, it also fails to resolve the weak support for several clades, such as H.sect.Ponerorchis (Tang et al. 2015), which may be one of the reasons why the affinities of the *Hemipilias. l.* are still controversial (Tang et al. 2015; Jin et al. 2017; Yang et al. 2022). In addition, the plastid tree constructed in this study shows slight differences from that of Yang et al. (2022), particularly in the clade comprising *H.yajiangensis* and *H.galeata*, which may be attributed to the differences in the partitioning model employed. H.sect.Hemipilia is a stable clade in phylogenetic analyses, and the discovery of *H.zhuxiensis* would prove to be significant in understanding phylogenetic relationships within *Hemipilia.* Moreover, *H.zhuxiensis* also provides morphological characteristics for defining the taxonomic boundary of H.sect.Hemipilia.

## Supplementary Material

XML Treatment for Hemipilia zhuxiensis

## ﻿Additional information

### Author ORCIDs

Cai-quan Shen https://orcid.org/0009-0009-9091-4650

Xi-Tang Chen https://orcid.org/0000-0002-5461-7183

De-Qing Lan https://orcid.org/0000-0003-3277-357X

Rui Qin https://orcid.org/0000-0003-4093-7044

Hong Liu https://orcid.org/0000-0001-7227-2476

### Data availability

All of the data that support the findings of this study are available in the main text.

## ﻿Appendix 1

**Table A1.** The information of all the herbarium specimens examined in this study. “*” represents self-collected specimens, “–” represents information loss.

The information of all the herbarium specimens			
Herbarium	Scientific Name	Collection Locality	Year
HIB 0256186	*Hemipiliazhuxiensis**	China, Hubei	2020
HIB 0256188	*Hemipiliahenryi**	China, Hubei	2020
PE 01517112	*Hemipiliahenryi*	China, Hubei	1977
PE 01517111	*Hemipiliahenryi*	China, Hubei	1977
PE 01517109	*Hemipiliahenryi*	China, Hubei	1938
PE 01056970	*Hemipiliahenryi*	China, Hubei	1976
PE 01056968	*Hemipiliahenryi*	China, Hubei	1976
PE 01056969	*Hemipiliahenryi*	China, Hubei	1976
PE 01056967	*Hemipiliahenryi*	China, Hubei	1976
PE 00340918	*Hemipiliahenryi*	China, Sichuan	1931
PE 00340917	*Hemipiliahenryi*	China, Sichuan	1931
PE 00340916	*Hemipiliahenryi*	China, Sichuan	1931
PE 00340915	*Hemipiliahenryi*	China, Sichuan	1931
PE 00340913	*Hemipiliahenryi*	China, Sichuan	1934
PE 00340914	*Hemipiliahenryi*	China, Sichuan	1931
PE 00340912	*Hemipiliahenryi*	China, Sichuan	1934
PE 00340911	*Hemipiliahenryi*	China, Sichuan	1934
PE 00340910	*Hemipiliahenryi*	China, Sichuan	1976
PE 00340909	*Hemipiliahenryi*	China, Chongqing	1958
PE 00340908	*Hemipiliahenryi*	China, Chongqing	1964
IBSC 0636166	*Hemipiliahenryi*	China, Hubei	1985
IBSC 0636169	*Hemipiliahenryi*	China, Guangxi	1939
IBSC 0636168	*Hemipiliahenryi*	China, Guangxi	1937
IBSC 0636165	*Hemipiliahenryi*	China, Sichuan	1979
IBSC 0636167	*Hemipiliahenryi*	China, Hubei	1985
KUN 1393999	*Hemipiliahenryi*	China, Hubei	2011
KUN 0023146	*Hemipiliahenryi*	China, Sichuan	1930
KUN 0023147	*Hemipiliahenryi*	China, Sichuan	1934
WUK 0350417	*Hemipiliahenryi*	China, Sichuan	1959
WUK 0138378	*Hemipiliahenryi*	China, Sichuan	1959
WUK 0239782	*Hemipiliahenryi*	China, Sichuan	1964
WUK 0330459	*Hemipiliahenryi*	China, Sichuan	1934
NAS NAS00558549	*Hemipiliahenryi*	China, Sichuan	1957
NAS NAS00560681	*Hemipiliahenryi*	China, Hubei	1978
SZ 00039926	*Hemipiliahenryi*	China, Sichuan	1964
SZ 00039927	*Hemipiliahenryi*	China, Sichuan	1954
CDBI CDBI0171211	*Hemipiliahenryi*	China, Sichuan	1976
CDBI CDBI0171210	*Hemipiliahenryi*	China, Sichuan	1976
CDBI CDBI0180069	*Hemipiliahenryi*	China, Sichuan	2003
N 050025209	*Hemipiliahenryi*	China, Hubei	1922
SYS SYS00028750	*Hemipiliahenryi*	China, Hubei	–
IMC IMC0012888	*Hemipiliahenryi*	China, Guizhou	2003
IMC IMC0012889	*Hemipiliahenryi*	China, Chongqing	2004
HUH GH00100247	*Hemipiliahenryi*	China	–
NY 00008940	*Hemipiliahenryi*	China	–
NY 00579391	*Hemipiliahenryi*	China	1901
P P00369342	*Hemipiliahenryi*	China	–
P P00259950	*Hemipiliahenryi*	China	–
NAS NAS00633540	*Hemipiliahenryi*	China, Hubei	2019
PE 02023829	*Hemipiliacrassicalcarata*	China, Henan	2009
PE 01681710	*Hemipiliacrassicalcarata*	China, Sichuan	1998
PE 01527207	*Hemipiliacrassicalcarata*	China, Sichuan	1998
PE 01517113	*Hemipiliacrassicalcarata*	China, Shaanxi	1959
PE 00340868	*Hemipiliacrassicalcarata*	China, Sichuan	1930
PE 00340867	*Hemipiliacrassicalcarata*	China, Sichuan	1984
PE 00340866	*Hemipiliacrassicalcarata*	China, Hubei	1956
PE 00340865	*Hemipiliacrassicalcarata*	China, Shaanxi	1952
PE 00340864	*Hemipiliacrassicalcarata*	China, Shaanxi	1959
PE 00340862	*Hemipiliacrassicalcarata*	China, Shanxi	1959
PE 00027177	*Hemipiliacrassicalcarata*	China, Sichuan	1928
PE 00340863	*Hemipiliacrassicalcarata*	China, Henan	1992
IBSC 0636150	*Hemipiliacrassicalcarata*	China, Hubei	1986
WUK 0059599	*Hemipiliacrassicalcarata*	China, Shaanxi	1952
WUK 0327685	*Hemipiliacrassicalcarata*	China, Shanxi	1959
HNWP 8224	*Hemipiliacrassicalcarata*	China, Shanxi	1959
SZ 00039897	*Hemipiliacrassicalcarata*	China, Shaanxi	1959
BNU 009588	*Hemipiliacrassicalcarata*	China, Shanxi	2014
HENU 0450489	*Hemipiliacrassicalcarata*	China, Henan	1978
HENU 0450490	*Hemipiliacrassicalcarata*	China, Henan	1978
HENU 0450491	*Hemipiliacrassicalcarata*	China, Henan	1978
HENU 0450492	*Hemipiliacrassicalcarata*	China, Henan	1978
HENU 0450493	*Hemipiliacrassicalcarata*	China, Henan	1978
GNUG GNUG0006147	*Hemipiliacrassicalcarata*	China, Guizhou	1993
CDCM CDCM0003656	*Hemipiliacrassicalcarata*	China, Sichuan	1978
E E00162723	*Hemipiliacrassicalcarata*	China, Sichuan	2003

“*” repersents self-collected specimen, “–” repersents information loss.
